# Influence of Sitting Positions and Level of Trunk Control During Reaching Movements in Late Preterm and Full-Term Infants

**DOI:** 10.3389/fped.2018.00185

**Published:** 2018-06-25

**Authors:** Natália T. da Silva Sato, Eloisa Tudella

**Affiliations:** Movement Analysis and Research Laboratory, Department of Physiotherapy, Federal University of São Carlos, São Paulo, Brazil

**Keywords:** independent sitting, trunk control, posture, reaching, premature, kinematics

## Abstract

In order to acquire reaching and independent sitting, refinement of trunk control is needed by gradually and progressively incorporating the head, thoracic, lumbar, and sacral segments. Previous studies have evaluated trunk control in a segmental way, standardizing the level of manual support in the infants' trunk during reaching. The aim of this study was to identify the level of trunk control and to analyze the influence of the difference sitting positions in late preterm and full-term infants between 6 and 8 months of age during reaching. Therefore, 36 infants born full term (control group)—FTG and 20 late preterm infants at a corrected age (experimental group)—PTG were evaluated. Most of the infants started the study at 6 months and they were evaluated monthly until 8 months of age (longitudinal study) in a total of 1–3 visits. The Segmental Assessment of Trunk Control was used to identify the level of trunk control in a segmental way, as well as to verify the capacity of the infant to maintain or regain the vertical position while sitting. Kinematic analysis was used for reaching. The infants were in a ring sitting position and at 90° of flexion. To elicit reaching, an attractive object was presented at the infant's midline and at 45° to the right and left. We found that PTG infants presented lower trunk control scores, i.e., worse control. For both groups, the ring sitting position and at 90° of flexion did not influence most kinematic variables during reaching because accurate manual support was provided for the infants' trunk. The PTG group presented less trunk displacement when at 90° of flexion. Compared to the FTG, even with accurate trunk support, the PTG group presented more immature reaches. These results suggest that accurate manual trunk support favored more stability of the trunk during the reach. Thus, early intervention is suggested for PTG infants and reaching in this age group should be trained in the ring sitting position with their trunk accurately manually supported. SATCo is an effective tool for segmental trunk evaluation.

## Introduction

The abilities to reach and sit independently are considered interrelated motor landmarks (1) as they are acquired during the first year of life. They are extremely important as they help infants to explore and interact with the environment (2). The development of reaching, however, depends on the interaction between intrinsic factors, for example postural control, as well as extrinsic factors, such as the conditions and experiences infants go through (3, 4). Infants at risk, such as premature infants may experience problems in reaching or delays in the ability to reach, hindering them to explore the environment in which they live (5–8). These changes in the ability to reach are probably due to the deficit in postural control caused by altered muscle tone (9), delayed coordination of trunk flexor, and extensor muscle activation, as well as top-down recruitment of the postural muscles (9).

Studies have shown that preterm infants with a gestational age of 32–36 weeks started reaching at a corrected age of around 4 ½ months (6, 7, 10, 11). In addition to this delay in reaching, there is also evidence that the frequency and quality of reaching in preterm infants is lower compared to full-term infants (6, 7, 12). According to a study carried out by Toledo and Tudella (13), late preterm infants (*M* = 35.6 weeks of gestational age) aged 6 and 7 months presented reaches with a higher adjustment index and a lower average and final velocity, which characterizes slow movements with more adjustments (13).

Reaching is influenced by the infant's capacity to control the sitting position (9, 14–17) through good postural control. The ability to maintain balance in the sitting posture gradually emerges in full-term infants between 2 and 9 months old (18). When infants are 5–6 months old, they are able to prop sit or sit independently for short periods (19). At 6 months, hip joint mobility increases, allowing the thighs to rest on the contact surface and the infant is able to sit in a ring position (20), i.e., symmetrically flexed, abducted, and externally rotated hips and flexed knees. At 7 months, infants begin to experiment new positions using lower limbs. Consequently, after being able to sit independently, infants learn to perform a coordinated action between upper limbs, trunk and lower limbs (14). Furthermore, at 8–9 months, the trunk and pelvis muscles stabilize the sitting position, allowing the infant to narrow the support base (1, 9, 21). The postural control of sitting may influence the development of reaching in atypically developing infants (22). Kyvelidou et al. (2) verified postural control at the emergence of sitting in infants born full-term, preterm with motor delays, and preterm who have a diagnosis of cerebral palsy. This finding suggests that infants with cerebral palsy presented a smaller center of pressure (COP) variability than preterm infants with motor delays, due to spasticity and stiffness, and thus, these infants may reduce the degrees of freedom to maintain stability during sitting. On the contrary, preterm infants with motor delays were delayed without presenting spasticity. Dusing et al. (23) verified if infants born preterm adapt their postural variability during the emergence of midline head control and initial reaching between 2.5 and 5 months of age by means of COP displacement. The finding suggests that preterm infants did not reduce postural variability (caudal cephalic direction) until 4 months of age, that is, they were not able to adapt their postural variability even after they had midline head control and were learning to reach. This result could mean that preterm infants have difficulty in stabilizing the trunk while focusing on head control or reaching.

Butler et al. (18) and Saavedra et al. (24) state that trunk control develops in the cephalocaudal direction and that neuromuscular coordination of the trunk occurs due to gradually and progressively incorporating the head, thoracic, lumbar, and sacral segments (18, 24). According to Butler et al. (18), the trunk is formed by many muscles and skeletal subunits, therefore it should be evaluated in segments rather than in blocks. Segmental Assessment of Trunk Control (SATCo) is a validated tool that aims to assess the level of trunk control in a segmental way, as well as to verify the capacity of the infant to maintain or regain the vertical position while sitting. SATCo, therefore, allows a thorough analysis of trunk control in typical and atypical infants, since until disseminating this assessment, the trunk was evaluated as a single unit. By using SATCo, we can identify the complete acquisition of trunk control in a segmental way. This makes it possible to identify delays in motor and sensory development and, thus to specifically rehabilitate the level of disability making intervention more effective (18).

Rachwani et al. (17) observed the influence of external thoracic and pelvic trunk support on reaching movements in typical 4–6 month old infants using kinematic analysis and SATCo. The infants were divided into 2 groups: (1) infants' trunk control level with score 4 and 5 (control of lower and upper thoracic trunk, respectively); (2) infants' trunk control level with score 6 and 7 (lower lumbar trunk control and total trunk control, respectively). The authors found that by providing thoracic support (below the angle of the scapula), the reaching performance was similar for infants in both groups. However, when providing pelvic support (around pelvis) for infants with thoracic control, more immature reaches were observed. When the pelvic support was provided for group 2, the reaches were more stable, rectilinear, and accurate and there was more trunk stability, shown by less displacement. Thus, the authors affirm that trunk control influences the quality of reaching (17).

In another study, Rachwani et al. (25) observed the effect of external upper trunk support (average thoracic region—below the angle of the scapula) and lower (lower lumbar region—pelvis) on the vertical sitting position during reaching in typical infants aged 2.5–8 months using kinematic analysis, electromyography and SATCo. The authors found that when infants were not sitting independently, the reaches were immature and postural instability was greater when they received pelvic support compared to the thoracic support. Concerning trunk instability, there was an increase in postural and upper limb muscle activity. However, external support (average thoracic region or lower lumbar region) did not influence the reaching performance when infants had already developed the ability to sit independently. The authors state that trunk control is acquired in a segmental way and is strongly correlated with better manual reaching performance (25).

Silva et al. (26) evaluated the influence of different sitting postures (ring and 90° flexion) on the reaching performance using kinematic analysis and SATCo in full-term infants aged 6–7 months. All the infants in the two sitting positions received manual pelvic support during the kinematic assessment of reaching. It was found that infants aged 6 months presented average thoracic control and that the ring sitting position favored more fluid and straighter reaches compared to the 90° of flexion position. In contrast, infants aged 7 months had high lumbar control and did not show any differences between sitting positions. The results obtained from this study confirm the need for efficient trunk control to provide stability during reaching (26).

Considering the above, a study is needed to evaluate the influence of accurate manual support in late preterm infants' trunks at a corrected age and at full term aged between 6 and 8 months during reaching in the different sitting positions (ring and 90° of flexion). This period is considered important in infants' motor development because it is the beginning of independent sitting for short periods of time and independent sitting having hands free to handle objects. It should be emphasized that, to the best of our knowledge, there are no studies on late preterm infants considering the trunk in a segmental way and its relationship with reaching in the different sitting positions (ring and 90° of flexion). In addition, in the three studies cited (17, 25, 26) which used SATCo and kinematics to verify the influence of trunk control during reaching, the authors pre-determined manual support, trunk, or pelvic regardless of the level of infants' trunk control. On the other hand, in our study, we offered manual support during the kinematic assessment of reaching at the accurate level of trunk control of each infant, according to SATCo. Thus, the following question can be raised: Do late preterm infants at a corrected age have less trunk control than full-term infants?

We believe that this study will contribute to new scientific evidence about the level of trunk control and its importance for late preterm infants during reaching in a sitting position. Having this information, therapists will be able to develop targeted intervention strategies, maximizing trunk control, and functionality of these infants' upper limbs.

Therefore, the aim of this study is to identify the level of trunk control of late preterm infants aged from 6 to 8 months at a corrected age and to analyze the influence of different sitting positions (ring and 90° of flexion) with the accurate manual support according to the level of trunk control (assessed by SATCo) during reaching.

We hypothesize that: (1) late preterm infants will present less trunk control than full-term infants, according to SATCo; (2) at 6 months old, late preterm and full-term infants will have a higher frequency of reaches in the ring sitting position when compared with the 90° of flexion sitting; (3) at 7–8 months old, late preterm and full term infants will have a higher frequency of reaches in the sitting position at flexion of 90° when compared with the ring position, and (4) when providing accurate trunk support (assessed by SATCo) to late preterm and full-term infants, the different sitting positions (ring and 90° of flexion) will have no influence on the kinematic variables of reaching.

## Materials and methods

### Participants

Thirty-six healthy, full-term infants (37–41 weeks and 6 days of gestational age) and 20 healthy late preterm infants (32 to < 37 weeks of gestational age) took part in this longitudinal study (27, 28). These infants were divided into 2 groups: (1) control group: full-term, and (2) experimental group: late preterm. The infants had to have an adequate birth weight for gestational age (27, 28) and an Apgar score from 7 to 10 in the first and 15 min. Full-term infants started the study with a mean age of 5.8 months (176.6 days ± *SD*: 6.3 days) and were evaluated once a month until they were 8 months old (231.6 days ± *SD*: 8.8 days). Among these 36 full-term infants: 17 were evaluated until 1 month, 11 were evaluated 2 months and 8 were evaluated 3 months. Late preterm infants started the study with a mean age of 6 months at a corrected age (211.7 days ± *SD*: 13.9 days) and were assessed monthly until they were 8 months old at a corrected age (273.8 days ± *SD*: 10.4 days). Among these 20 late preterm infants: 11 were evaluated until 1 month, 6 were evaluated 2 months and 3 were evaluated 3 months. Infants with neurosensory motor or musculoskeletal changes were not included in the study.

The infants were recruited from a maternity ward and from Basic Health Units in a medium-sized city in the interior of São Paulo state (SP, Brazil). This study was carried out in accordance with the recommendations of Guidelines and Norms Regulating Research Involving human subjects (Resolution 466/2012, of the National Health Council, protocol number 1.350.978, Research Ethics Committee of the Federal University of São Carlos (CEP/UFSCar), with written informed consent from all the subjects. All subjects gave written informed consent in accordance with the Declaration of Helsinki. The protocol was approved by the Research Ethics Committee of UFSCar (CEP/UFSCar).

### Materials and procedures

In the first evaluation, the parents and/or guardians were informed again about the procedures and objectives of the study. If they agreed, they signed the Free and Informed Consent Term and answered the questionnaire regarding the information about pregnancy, delivery and the infant's health. During the monthly evaluations, all the infants were clinically tested using the SATCo (18) to determine the level of trunk control and the Alberta Infant Motor Scale—AIMS (29) to ensure that full-term infants had a minimum percentile of 25. The kinematic analysis was used to analyze reaching.

### Segmental assessment of trunk control

The evaluation consists of providing manual support with the therapist's hands positioned firmly and horizontally on various anatomical landmarks of the infant's trunk. Manual support starts at the shoulders to evaluate the cervical control (head), followed by the axilla (upper thoracic control), lower scapula (mean thoracic control), above the lower ribs (lower thoracic control), below the ribs (upper lumbar control), pelvis (lower lumbar control), and without support (total trunk control). During each level of manual support, three important aspects of trunk control are evaluated: (1) Static control: infant's ability to maintain a neutral trunk posture vertically for 5 s; (2) Active control: infant's ability to maintain a neutral trunk posture during head movements, and (3) Reactive control: infant's ability to maintain or regain trunk control in a controlled and balanced way following a threat to balance, produced by a brisk nudge on the right and left acromion, in the xiphoid process and in the infant's 7th cervical vertebra. If the infant maintains trunk control in the three equilibrium tests at the assessed level, the test continues by reducing the level of manual support until the infant cannot maintain the initial, upright, and balanced posture (18). The ability of the infant to maintain or regain the vertical position at the different trunk levels is evaluated by the presence or absence of control during static, active and reactive testing. The score reflects the region in which the infant is able to maintain trunk control: score (1): head control, (2): upper thoracic, (3): mid thoracic, (4): lower thoracic, (5): upper lumbar, (6): lower lumbar (7): total trunk control (18).

To identify the level of trunk control, the researcher sat behind the infant with her hands positioned horizontally around the infant's trunk, mentioned in SATCo. The second evaluator sat in front of the infant and presented him/her with attractive objects in order to hold his attention and keep the upper limbs of the infant high. The examiner/assessor also helped by nudging in the reactive test. All evaluations were conducted by the same evaluators. The examiners had been trained in the previous data collection period of infants who did not participate in the study. The level of trunk control was assessed using SATCo scores for each infant. For SATCo, the inter-rater agreement was 90% calculated for 20% of the total sample using the equation: number of agreements/(number of agreements + number of disagreements) × 100.

In this study, the level of trunk control was considered complete when the presence of control was recorded in the 3 equilibrium tests, i.e., static, active, and reactive. If the infant presented control only in the static test, or static and active test, the level of the trunk control considered was the previous one to what was being tested.

### Reach kinematics

After carrying out the SATCo assessment, reaching was evaluated using the Qualisys® system. To do this, four reflexive passive spherical markers (12 mm diameter) were fixed to the head (midline of the frontal bone) the trunk (height of the middle portion of the sternum), and the wrists (between the radial and ulnar styloid process) of the infants. In addition, in order to differentiate the infant's upper limb movement from the target movement, a reflective marker was placed on the central upper part of the object to be offered to the infant.

### Test procedures

**Figure 1:**
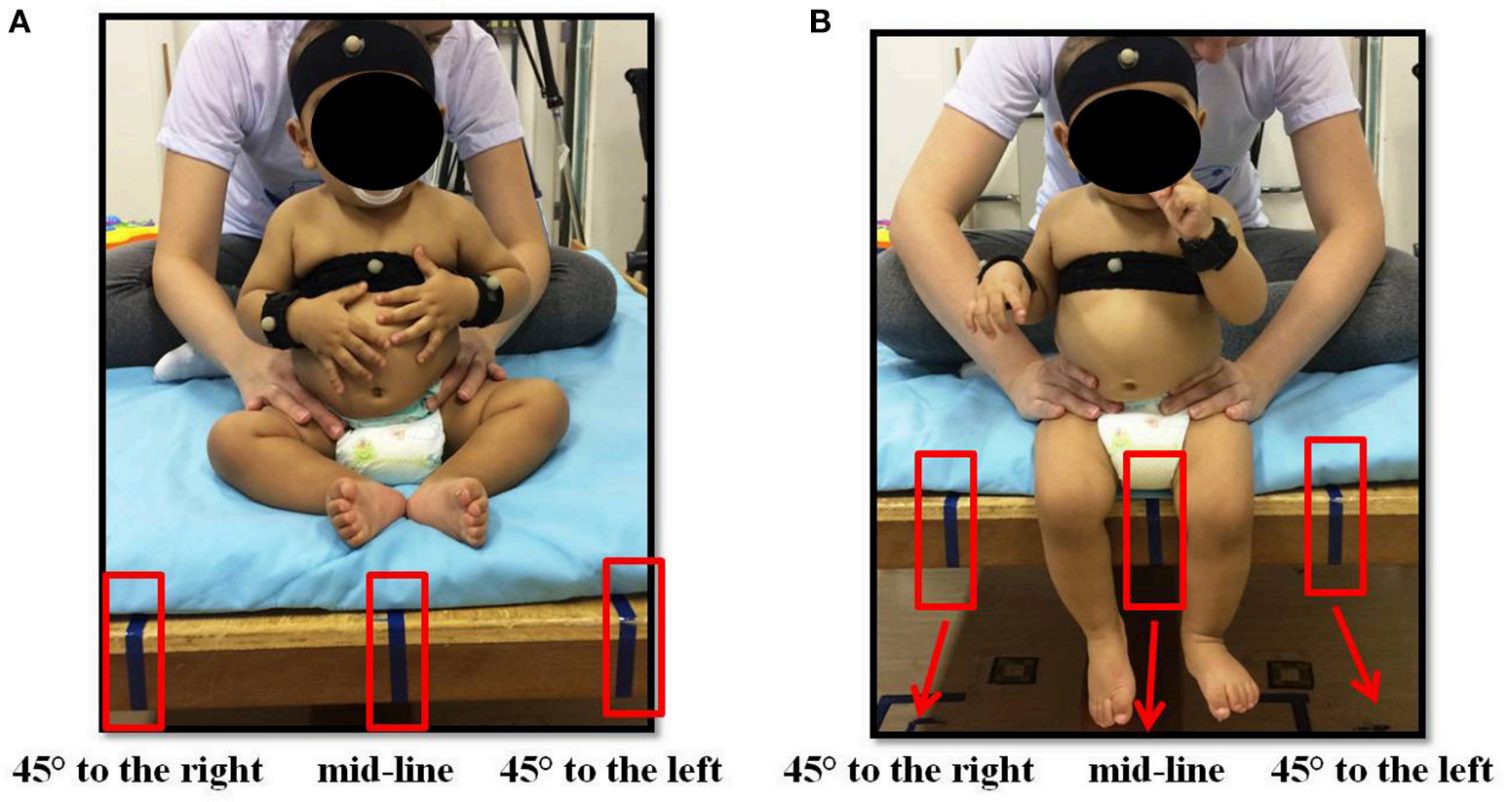
**(A)** Infant sitting in ring position (mid-line: the object was presented in the midline of the infant's body, at the level of xiphoid process; 45° to the right and 45° to the left: the object was presented approximately 45° from the midline, at the level of xiphoid process); **(B)** infant sitting at a flexion of 90° (mid-line: the object was presented in the midline of the infant's body, at the level of xiphoid process; 45° to the right and 45° to the left: the object was presented ~45° from the midline, at the level of xiphoid process.

To carry out the kinematic assessment of reaching, infants were positioned on a wooden bench in the ring position (Figures 1A,B) and at 90° of flexion of hips, knees and ankles (Figures 1A,B). The evaluation sequences of the sitting positions were alternated (ring and 90° of flexion) and the different directions that the researcher presented the object to the infant (45° to the right, 45° to the left, and mid-line) were alternated from one infant to another, i.e., half of the infants started the assessment in the ring sitting position and the other half began the assessment in the sitting position at 90° of flexion. The same procedure was followed when the object was presented to the infant.

While the infant remained seated, a researcher sitting behind the infant provided accurate manual support to the trunk, according to the level of trunk control in the SATCo. To elicit reaching, another researcher, who sat in front of the infant, presented one rigid (rattle) or one malleable (natural rubber toy) object in the midline of the infant's body at the level of xiphoid process in front of the infant at the distance corresponding to the length of the child's upper limb (30), and to the right and left, approximately 45° from the midline (Figures 1A,B).

The researcher called the infant's attention to the object, moving it momentarily so that the infant could perceive it and reach it. The exposure time of the object was 40 s (timed by the Qualisys System) in each direction (midline, 45° to the right, and 45° to the left), and between each successful reach, it was taken away and put back at an interval of 2 s. If the infant did not touch the object, it was removed and shown again. First, the rigid object was presented. If the infant did not show interest in the object, it was presented in another color, or as a second option, the rigid object was replaced by the malleable object. The total time of the evaluation was 2 min in each sitting position, making a total of 4 min of kinematic assessment. All evaluations were conducted by the same evaluators. Both evaluators analyzed the spatio-temporal variables of reaching and were trained to identify and nominate each marker placed on the infant and to identify, frame by frame, the beginning and end of the reaching movement. The examiners were trained prior to initiating the data collection in infants who did not participate in the study. The examiners were not blind to the infants' gestational age. Only the hand that first touched the toy was considered for kinematics analyses. The inter-rater agreement, assessed by the equation: number of agreements/(number of agreements + number of disagreements) × 100, was 97.46% from 20% of the total sample, considering the frequency of reaching, that is, the initial and final frame of each reaching.

### Data analysis system

Reaching was considered valid when the infant's hand came into contact with the toy (31), when the reaching infant was visually turned toward the toy and performed the movement with one or both upper limbs toward the target until touching it, with or without grasping (32, 33). The beginning of a reach was defined as the first frame when the infant's arm began an uninterrupted movement, leaving the midline of the waist or below, toward the target. The end of a reach was defined as the first frame when the infant's hand touched the target (7, 30, 31, 34). In the reaches performed with both hands, only the hand that touched the object first was analyzed.

The Kinovea 0.8.21 software was used to analyze the frequency of reaches and observe the first and last frames of reaching. To record quantitative kinematic analysis data of reaching in infants, the Qualisys Motion Capture System (Qualisys AB, 411 12 Gothenburg, Sweden) was adopted. In the experiment, five cameras (Qualisys Oqus 300) were used: four for quantitative analysis and one for qualitative analysis capturing images at a frequency of 200 Hz. These cameras were coupled to a 2.8 GHz Pentium 4 computer which records the data and analyzes the three-dimensional information of the movement using Qualisys Track Manager 2.6 (QTM) software. Frame-by-frame identification of the beginning and end of reaching was carried out using the QTM software and these files were exported in TSV format and saved on DVDs. Afterwards, MATLAB R2013a (8.1.0.604) was used to apply the 2nd order digital Butterworth filter with a cut-off frequency of 6 Hz. Based on the MATLAB routines, the values of the spatial and time kinematic variables were calculated on the right, on the left, and at the midline (movement unit, straightness index, movement duration, mean velocity, deceleration index, peak velocity, and trunk displacement).

### Description of dependent variables of reaching

Frequency of reaching: this was calculated as the number of reaches considered valid (when the infant's arm began an uninterrupted movement, leaving the midline of the waist or below, and touched the toy) during the 40 s period in each direction (45° to the right, 45° to the left and midline); in total 2 min in each sitting position.

Spatial and temporal variables: movement unit (MU) was defined as the maximum velocity (peak) between two minimum velocities (valley), the difference being greater than 1 cm/s, that is, the lower the number of movement units, the better the reaching movement (8, 35); the straightness index (SI) was calculated by the ratio between the shortest distance that can be traversed in this trajectory by the actual distance traversed by the hand. It indicates how straight the trajectory of the movement is. The closer to 1 the index is, the more rectilinear the movement is (8, 34, 35); deceleration index (DI) was calculated by the ratio between the time of the movement after the highest velocity peak and the total duration of reaching multiplied by 100. The higher this variable, the longer the time spent on decelerating the movement of the upper limb (8, 36); movement duration (MD) was given by the time difference between the end and the beginning of the reaching, that is, the shorter the duration, the faster the movement (36, 37); mean velocity (MV) was obtained by the ratio between the distance traversed by the hand and the time spent on the movement (8, 36, 37); peak velocity (PV) consists of the maximum speed reached during the movement of the upper limb (35), the shorter the peak velocity, the better the quality of the movement (16) and trunk displacement (TD) is the distance covered by the sternal marker, calculated by the sum of distances covered in the three movement axes, the lower the trunk displacement, the lower the trunk mobility (26).

### Statistical analysis

Inferential procedures for testing homogeneity (Levene test) and normality of variances (Shapiro-Wilk and Kolmogorov-Smirnov tests) were used. Descriptive analysis was made using percentages to identify the level of trunk control in the FTG and PTG. The frequency of reaching was analyzed by the mean frequency of occurrence in each group (full-term and late preterm), in each visit (age – 6, 7, and 8 months), in each posture (ring and flexion of 90°) and each of the three directions (45° to the right, 45° to the left, and midline). The frequency variables of reaching to the right, left, and midline and the movement unit to the right were analyzed using the Wilcoxon test to compare the sitting positions in each group (control and experimental) and the Mann-Whitney test to compare the groups (control and experimental). The spatio and temporal variables were analyzed using the mean values of reaching performed in each direction (45° to the right, 45° to the left, and midline), in each posture (ring and 90° of flexion) for each assessment carried out. The Mixed Linear Model and sequential Bonferroni adjustment application were used for the multiple comparisons. The factors considered in each reaching direction were: group (full-term and late preterm), time (visits or ages – 6, 7, and 8 months), posture (ring and 90° of flexion), and interaction among these components (group^*^time^*^posture; time^*^group; time^*^posture; and group^*^posture). For all tests, the level of significance was α < 0.05. We used Cohen's d for parametric tests to calculate effect sizes (*d* < 0.2, small effect; *d* > 0.2 and < 0.5, moderate effect; *d* > 0.5, large effect), and Z scores generated in the analyses were used to calculate effect sizes (*r* < 0.2: small effect; *r* > 0.2 and < 0.4: moderate effect; *r* > 0.5, large effect to non-parametric tests. The analyses were carried out using the Statistical Package for the Social Sciences (SPSS) software version 20.0.

## Results

The aim of this study was to identify the level of trunk control in all the infants assessed and to verify the influence of different sitting positions (ring and 90° of flexion) with the accurate manual support for the infant's trunk during reaching of late preterm infants at corrected age and full-term infants.

### Identifying level of trunk control by group and time

It can be observed in Table 1 that the trunk control presented a progressive and descending order (cephalocaudal) with increasing age in both groups. In addition, late preterm infants at corrected age were found to have lower trunk control compared to full-term infants at all ages. At 6 months old, it was observed that 9.09% of the late preterm infants were still at level 1 of trunk control (cervical control), whereas in full-term infants the lowest control presented was level 2 (upper thoracic control). Still at this age, 45.45% of the late preterm infants presented levels 2 and 3 of trunk control (upper thoracic and mid thoracic control), while 60% of full-term infants had a trunk control of level 4 (lower thoracic control). At 7 months old, it was observed that the level of trunk control varied between late preterm infants and full-term infants. However, in late preterm infants, 18.18% presented level 5 (upper lumbar control), while in full-term infants, 9.09% presented level 7 (total trunk control). At 8 months old, in the late preterm group, it is interesting to note that only 10% presented total trunk control, while in the full-term group, 71.42% of the infants had control at this same level.

**Table 1:** Percentage of level of trunk control by group and age.

**SATCo**		**% Full term**			**% Late preterm**		
**Score**	**Functional level**	**6 M**	**7 M**	**8 M**	**6 M**	**7 M**	**8 M**
1	Cervical control	–	–	–	9.09	–	–
2	Upper thoracic	10	–	–	45.45	9.09	10
3	Middle thoracic	30	13.63	–	45.45	36.36	10
4	Lower thoracic	60	27.27	–	–	36.36	10
5	Upper lumbar	–	36.36	4.76	–	18.18	20
6	Lower lumbar	–	13.63	23.81	–	–	40
7	Full trunk control	–	9.09	71.42	–	–	10

*The dependent variables are expressed as percentages (%), M, months*.

### Frequency of reaching

A total number of 1,384 reaches were considered valid: 874 performed by full-term infants (control group) and 510 by late preterm infants (experimental group) at a corrected age.

#### Frequency of reaching—intra-group comparison

It can be observed in Table 2 that there was no significant difference when comparing the sitting positions (ring and flexion) at each visit (6, 7, and 8 months) and each group (full-term and late preterm). The effect size was reported in the same table.

**Table 2:** Comparison between positions (ring vs. flexion) in each group (full-term and late preterm) and in each age (6, 7, and 8 months) of the frequency of reaching in each direction (45° to the right, 45° to the left and midline) (Wilcoxon test).

**Variable**	**Posture**	**Full term–Control group**						**Late preterm–Experimental group**					
		**6 M**		**7 M**		**8 M**		**6 M**		**7 M**		**8 M**	
		***p***	***r***	***p***	***r***	***p***	***r***	***p***	***r***	***p***	***r***	***p***	***r***
Freq. R	Ring	0.95	−0.02^*^	0.56	−0.07^*^	0.50	0.06^*^	0.09	0.35^**^	0.58	0.14^*^	0.12	0.25^**^
	Flexion												
Freq. L	Ring	0.67	0.00^*^	0.40	0.15^*^	0.43	0.00^*^	0.67	0.00^*^	0.08	−0.40^**^	0.41	−0.11^*^
	Flexion												
Freq. ML	Ring	1.00	0.03^*^	0.26	−0.10^*^	0.91	0.16^*^	1.00	0.03^*^	0.60	0.00^*^	0.54	0.07^*^
	Flexion												

Mean frequency of occurrence in each visit (age), in each group (full-term and late preterm) and each of the three direction. Freq. R, frequency to the right; Freq. L, frequency to the left; Freq.ML, frequency at midline; M, months; p: significance level to 6, 7, and 8 months in each group, in each position; ^*^Significant difference (value p); r, effect size (calculate r using means and standard deviations);

* *effect size small*;

** *effect size medium*.

#### Frequency of reaching—inter-group comparison

Table 3 shows a significant difference only in the comparison between groups (full-term and late preterm) in the ring sitting position at 7 months (*U* = 31.00; *p* < 0.01; *r* = 0.58) at 45° to the left direction. The effect size was reported in the same table.

**Table 3:** Comparison between groups (full-term vs. preterm) in each posture (ring and flexion) and each age (6, 7, and 8 months) of the frequency of reaching in each direction (45° to the right, 45° to the left and midline) (Mann-Whitney test).

**Variables**	**Posture**	**6 M**		**7 M**		**8 M**	
		***p***	***r***	***p***	***r***	***p***	***r***
Freq. R	Ring	0.2	−0.16^*^	0.6	−0.11^*^	0.1	−0.35^**^
	Flexion	0.9	0.12^*^	0.8	0.09^*^	0.3	−0.16^*^
Freq. L	Ring	0.8	0.09^*^	0.0^*^	0.58^***^	0.7	−0.06^*^
	Flexion	0.7	0.08^*^	0.3	0.12^*^	0.3	−0.18^*^
Freq. ML	Ring	0.2	−0.18^*^	0.5	0.15^*^	0.6	−0.15^*^
	Flexion	0.2	−0.23^**^	0.2	0.18^*^	0.1	−0.32^**^

Mean frequency of occurrence in each visit (age), in each position (ring and flexion) and each of the three directions. Freq. R, frequency to the right; Freq. L, frequency to the left; Freq.ML, frequency at midline; M, months; p, significance level; ^*^Significant difference to 6, 7, and 8 months in each position; r, effect size (calculate r using means and standard deviations);

* *effect size small*;

** *effect size medium*;

*** *effect size large*.

### Spatio and temporal variables

The meaningful results and the effect size for the spatio and temporal variables (number of movement units, straightness index, deceleration index, movement duration, mean velocity, peak velocity, and trunk displacement) in each of the 3 reaching directions (45° to the right, 45° to the left, and midline) are presented below (Table 4).

**Table 4:** Comparison between groups (full-term vs. preterm) in each posture (ring and flexion) and each age (6, 7, and 8 months)—mixed linear model for parametric tests and effect sizes of inter-groups (full-term vs. late preterm) (calculate using means and standard deviations) of all spatio-temporal variable, in each position (ring and flexion) and in each visit (age 6, 7, and 8 months).

**Variable**	**Postures**	**6 M**	**7 M**	**8M**
MU_ R	Ring	*p* = 0.14; *r* = −0.31^**^	*p* = 0.15; *r* = 0.25^**^	*p* = 0.76; *r* = −0.04^*^
	Flexion	*p* = 0.16; *r* = −0.31^**^	*p* = 0.06; *r* = −0.35^**^	*p* = 0.86; *r* = 0.00^*^
MU_ L	Ring	*p* = 0.04^*^; *d* = −0.62^***^	*p* = 0.73; *d* = −0.62^***^	*p* = 0.87; *d* = 0.14^*^
	Flexion	*p* = 0.11; *d* = −0.22^**^	*p* < 0.01^*^; *d* = −0.^***^	*p* = 0.81; *d* = 0.00^*^
MU_ ML	Ring	*p* = 0.11; *d* = 0.00 ^*^	*p* = 0.03^*^; *d* = −1.07^***^	*p* = 0.52; *d* = 0.^**^
	Flexion	*p* = 0.10; *d* = −0.49^**^	*p* = 0.02^*^; *d* = −0.^*^	*p* = 0.45; *d* = 0.63^***^
SI _R	Ring	*p* = 0.47; *d* = 0.63^***^	*p* = 0.15; *d* = 1.00^***^	*p* = 0.29; *d* = 0.00^*^
	Flexion	*p* = 0.76; *d* = 0.00^*^	*p* = 0.16; *d* = 0.00^*^	*p* = 0.94; *d* = 0.00^*^
SI_ L	Ring	*p* = 0.11; *d* = 0.99^***^	*p* = 0.46; *d* = 0.00^*^	*p* = 0.39; *d* = 0.63^***^
	Flexion	*p* = 0.06; *d* = 0.49^**^	*p* = 0.08; *d* = 1.00^***^	*p* = 0.59; *d* = 1.00^***^
SI_ ML	Ring	*p* = 0.23; *d* = 0.00^*^	*p* = 0.08; *d* = 0.99^***^	*p* = 0.90; *d* = 0.00^*^
	Flexion	*p* = 0.61; *d* = 0.99^***^	*p* = 0.35; *d* = 0.17^*^	*p* = 0.63; *d* = 0.00^*^
DI_R	Ring	*p* = 0.82; *d* = −0.09^*^	*p* = 0.45; *d* = −0.28^**^	*p* = 0.35; *d* = −0.39^**^
	Flexion	p < 0.01^*^; *d* = −1.70^***^	*p* = 0.54; *d* = −0.21^**^	*p* = 0.04^*^; *d* = −0.64^***^
DI_L	Ring	*p* = 0.21; *d* = 0.47^**^	*p* = 0.36; *d* = −0.35^**^	*p* = 0.02^*^; *d* = −0.98^***^
	Flexion	*p* = 0.45; *d* = 0.26^**^	*p* = 0.06; *d* = −0.67^***^	*p* = 0.22; *d* = −1.37^***^
DI_ML	Ring	*p* = 0.03^*^; *d* = −0.96^***^	*p* = 0.76; *d* = −0.13^*^	*p* = 0.09; *d* = −0.57^***^
	Flexion	*p* = 0.26; *d* = 0.48^**^	*p* = 0.70; *d* = 0.14^*^	*p* = 0.08; *d* = −0.72^***^
MD_R	Ring	*p* = 0.02^*^; *d* = −0.98^***^	*p* = 0.02^*^; *d* = −0.90^***^	*p* = 0.67; *d* = 0.33^**^
	Flexion	*p* = 0.04^*^; *d* = −0.86^***^	*p* < 0.01^*^; *d* = 0.00^*^	*p* = 0.58; *d* = 0.15^*^
MD_L	Ring	*p* = 0.03^*^; *d* = −0.66^***^	*p* = 0.69; *d* = −0.16^*^	*p* = 0.89; *d* = 0.00^*^
	Flexion	*p* = 0.35; *d* = 0.00*	*p* < 0.01^*^; *d* = −1.02^***^	*p* = 0.85; *d* = 0.28^**^
MD_ML	Ring	*p* = 0.50; *d* = −0.22^**^	*p* = 0.05; *d* = −0.97^***^	*p* = 0.56; *d* = 0.31^**^
	Flexion	*p* = 0.09; *d* = −0.49^**^	*p* < 0.01^*^; *d* = −1.05^***^	*p* = 0.26; *d* = 0.47^**^
MV_R	Ring	*p* = 0.04^*^; *d* = 0.85^***^	*p* = 0.93; *d* = −0.10^*^	*p* = 0.17; *d* = −0.56^***^
	Flexion	*p* = 0.02^*^; *d* = 0.76^***^	*p* = 0.03^*^; *d* = 0.78^***^	*p* = 0.07; *d* = −0.88^***^
MV_L	Ring	*p* = 0.15; *d* = 0.53^***^	*p* = 0.19; *d* = 0.41^**^	*p* < 0.01^*^; *d* = −1.44^***^
	Flexion	*p* = 0.13; *d* = 0.69^***^	*p* = 0.14; *d* = 0.60^***^	*p* = 0.57; *d* = −0.21^**^
MV_ML	Ring	*p* = 0.13; *d* = 0.66^***^	*p* = 0.37; *d* = 0.23^**^	*p* = 0.61; *d* = −0.10^*^
	Flexion	*p* = 0.07; *d* = 1.11^***^	*p* = 0.03^*^; *d* = 0.64^***^	*p* = 0.15; *d* = −0.62^***^
PV_R	Ring	*p* = 0.28; *d* = 0.42^**^	*p* = 0.89; *d* = −0.18^*^	*p* = 0.13; *d* = −0.60^***^
	Flexion	*p* = 0.10; *d* = 0.78^***^	*p* = 0.59; *d* = 0.31^**^	*p* = 0.09; *d* = −0.59^***^
PV_L	Ring	*p* = 0.88; *d* = 0.03^*^	*p* = 0.09; *d* = 0.57^***^	*p* = 0.02^*^; *d* = −1.02^***^
	Flexion	*p* = 0.19; *d* = 0.60^***^	*p* = 0.16; *d* = 0.61^***^	*p* = 0.74; *d* = 0.21^**^
PV_ML	Ring	*p* = 0.58; *d* = 0.37^**^	*p* = 0.88; *d* = 0.06^*^	*p* = 0.95; *d* = 0.10^*^
	Flexion	*p* = 0.60; *d* = 0.12^*^	*p* = 0.63; *d* = 0.15^*^	*p* = 0.38; *d* = −0.54^***^
TD_R	Ring	*p* = 0.41; *d* = 0.32^**^	*p* = 0.60; *d* = 0.57^***^	*p* = 0.05; *d* = 0.66^***^
	Flexion	*p* = 0.02^*^; *d* = 0.99^***^	*p* < = 0.01^*^; *d* = 1.73^***^	*p* < 0.01^*^; *d* = 1.40^***^
TD_L	Ring	*p* = 0.67; *d* = 0.10^*^	*p* = 0.52; *d* = 0.25^**^	*p* = 0.53; *d* = −0.06^*^
	Flexion	*p* = 0.73; *d* = 0.28^**^	*p* = 0.59; *d* = 0.47^**^	*p* = 0.44; *d* = 0.33^**^
TD_ML	Ring	*p* = 0.09; *d* = 0.89^***^	*p* = 0.12; *d* = 0.83^***^	*p* = 0.03^*^; *d* = 0.97^***^
	Flexion	*p* = 0.09; *d* = 0.76^***^	*p* < 0.01^*^; *d* = 1.22^***^	*p* = 0.02^*^; *d* = 1.05^***^

Cohen's d for parametric tests and effect size r for nonparametric tests. MU_R, movement unit direction toward the target on the right; MU_L, movement unit on the left; MU_ML, movement unit at midline; SI_R, straightness index to the right; SI_L, straightness index to the left; SI_ML, straightness index at midline; DI_R, deceleration index to the right; DI_L, deceleration index to the left, DI_ML, deceleration index at the midline; MD_R, movement duration to the right; MD_L, movement duration to the left; MD_ML, movement duration at midline; MV_R, mean velocity to the right; MV_L, mean velocity to the left; MV_ML, mean velocity at midline; PV_R, peak velocity to the right; PV_L, peak velocity to the left; PV_ML, peak velocity at midline; TD_R, trunk displacement to the right; TD_L, trunk displacement to the left; TD_ML, trunk displacement at midline; 6M, 6 months; 7M, 7 months; 8M, 8 months; p, significance level; ^*^Significant difference to 6, 7, and 8 months in each position; r, effect size (calculate r using means and standard deviations

* *effect size small*;

** *effect size medium*;

*** *effect size large*.

### Number of movement units

In the left direction, there was a significant difference in the group factor [*F*_(1, 146)_ = 5.69, *p* < 0.01; *d* = −0.20]. When comparing the groups, it was observed that the late preterm group had more movement units (*p* < 0.01; *d* = −0.20). In the midline direction, there was a significant difference between the interaction components: Group ^*^Time [*F*_(2, 149)_ = 4.02, *p* = 0.02]. The late preterm group had more movement units compared to the full-term group at 7 months old (*p* < 0.01; *d* = −0.92).

However, in the left direction, there was a large effect size in the ring position at 6 months (*d* = −0.62), and in the flexion position at 7 months (*d* = −0.85). In the midline direction, there was a large effect size in the ring position at 7 months (*d* = −1.07), and in the flexion position at 8 months (*d* = 0.63) (Table 4).

### Straightness index

In the left direction, there was a significant difference in the group factor [*F*_(1, 146)_ = 5.71, *p* < 0.01] and time [*F*_(2, 146)_ = 7.41, *p* < 0.01]. When comparing the groups, it was observed that the late preterm group had a lower straightness index (*p* < 0.01; *d* = 0.34). Regardless of the group, a lower straightness index was observed at 6 months compared to 7 months (*p* < 0.01; *d* = –0.62) and 8 months (*p* < 0.01; *d* = −0.58).

However, in the right direction there was a large effect size in the ring position at 6 and 7 months (*d* = 0.63, *d* = 1.00, respectively). In the left direction, there was a large effect size in the ring position at 6 and 8 months (*d* = 0.99, *d* = 0.63, respectively), and in the flexion position at 7 and 8 months (*d* = 1.00). In the midline direction, a large effect size was observed in the ring position at 7 months (*d* = 0.99), and in the flexion position at 6 months (*d* = 0.99) (Table 4).

### Deceleration index

In the right direction, there was a significant difference in the group factor [*F*_(1, 150)_ = 10.54, *p* < 0.01; *d* = −0.51]. When comparing the groups, it was observed that the late preterm group had a higher deceleration index (*p* < 0.01; *d* = −0.51). In the left direction, there was a significant difference between the interaction components Group^*^Time [*F*_(2, 146)_ = 5.55, *p* < 0.01; *d* = −1.15]. The late preterm group had a higher deceleration index compared to the full-term group at 8 months old (*p* < 0.01; *d* = −1.15).

However, in the left direction, there was a large effect size in the ring position at 8 months (*d* = −0.98), and in the flexion position at 7 and 8 months (*d* = −0.67, *d* = −1.37, respectively). In the midline direction, a large effect size was observed in the ring position at 6 and 8 months (*d* = −0.96, *d* = −0.57, respectively), and in the flexion position at 8 months (*d* = −0.72) (Table 4).

### Movement duration

In the right direction, there was a significant difference between the interaction components Group^*^Time to the right [*F*_(2, 150)_ = 5.58, *p* < 0.01]. When comparing the groups, it was observed that the late preterm group had a longer movement duration at 6 months (*p* < 0.01; *d* = −0.91), and 7 months (*p* < 0.01; *d* = −0.98). In the left direction, there was a significant difference in the group factor [*F*_(1, 146)_ = 5.84, *p* < 0.01; *d* = −0.30], in which the late preterm group had a longer movement duration. In the midline direction, there was also a significant difference between the interaction component Group^*^Time [*F*_(2, 149)_ = 5.16, *p* < 0.01; *d* = −0.97]. The late preterm group showed a longer movement duration at 7 months old (*p* < 0.01; *d* = −0.97).

However, in the right direction there was a large effect size in the ring position at 6 and 7 months (*d* = −0.98, *d* = −0.90, respectively), and in the flexion position at 6 months (*d* = −0.86). In the left direction, there was a large effect size in the ring position at 6 months (*d* = −0.66), and in the flexion position at 7 months (*d* = −1.02). In the midline direction, a large effect size was observed in the ring position at 7 months (*d* = −0.97) and in the flexion position at 7 months (*d* = −1.05) (Table 4).

### Mean velocity

In the right direction, there was a significant difference between the interaction component Group^*^Time [*F*_(2, 150)_ = 7.44, *p* < 0.01]. When comparing the groups, it was observed that the late preterm group had a lower mean velocity at 6 months old (*p* < 0.01; *d* = 0.85) and a higher mean velocity at 8 months old (*p* = 0.02; *d* = −0.67). In the left direction, there was a significant difference between the interaction component Group^*^Time [*F*_(2, 146)_ = 8.33, *p* < 0.01]. The late preterm group presented a lower mean velocity at 6 months (*p* = 0.03; *d* = 0.64), and 7 months (*p* = 0.05; *d* = 0.51) and a higher mean velocity at 8 months (*p* < 0.01; *d* = –0.84). In the midline direction, there was a significant difference between the interaction components Group^*^Time [*F*_(2, 149)_ = 4.30, *p* < 0.01], in which the late preterm group presented a lower mean velocity at 6 months (*p* = 0.02; *d* = 0.87), and 7 months (*p* = 0.03; *d* = 0.42).

However, in the right direction there was a large effect size in the ring position at 6 and 8 months (*d* = 0.85, *d* = −0.56, respectively), and in the flexion position at 6, 7, and 8 months (*d* = 0.76, *d* = 0.78, *d* = −0.88, respectively). In the left direction, there was a large effect size in the ring position at 6 and 7 months (*d* = 0.66, *d* = −1.44, respectively), and in the flexion position at 6 and 7 months (*d* = 0.69, *d* = 0.60 respectively). In the midline direction, a large effect size was observed in the ring position at 6 months (*d* = 0.66), and in the flexion position at 6, 7 and 8 months (*d* = 1.11, *d* = 0.64, *d* = −0.62, respectively) (Table 4).

### Peak velocity

In the right direction, there was a significant difference in the position factor [*F*_(1, 150)_ = 4.67, *p* = 0.03; *d* = 0.30]. When comparing the groups, the sitting position at 90° of flexion showed a lower peak velocity compared to the ring sitting position (*p* = 0.03; *d* = 0.30). There was also a significant difference between the interaction component Group^*^Time [*F*_(2, 150)_ = 4.39, *p* < 0.01], in which the late preterm group had a higher peak velocity at 8 months (*p* = 0.02; *d* = −0.60). In the left direction, there was a significant difference between the interaction component Group^*^Time [*F*_(2, 146)_ = 4.46, *p* < 0.01]. The late preterm group had the lowest peak velocity at 7 months (*p* = 0.03; *d* = 0.60); and a higher peak velocity at 8 months (*p* = 0.05; *d* = −0.52). In the midline direction, there was a significant difference between the interaction components Time^*^Position [*F*_(2, 149)_ = 3.17, *p* = 0.04]. The sitting position at 90° of flexion showed a lower peak velocity at 8 months compared to 6 months (*p* = 0.03; *d* = 0.95). There was a lower peak velocity in the ring sitting position compared to the sitting position at 90° of flexion at 6 months (*p* = 0.02; *d* = 0.63).

However, in the right direction there was a large effect size in the ring position at 8 months (*d* = −0.60), and in the flexion position at 6 and 8 months (*d* = 0.78, *d* = −0.59 respectively). In the left direction, there was a large effect size in the ring position at 7 and 8 months (*d* = 0.57, *d* = −1.02, respectively), and in the flexion position at 6 and 7 months (*d* = 0.60, *d* = 0.61, respectively). In the midline direction, a large effect size was observed in the flexion position at 8 months (*d* = −0.54) (Table 4).

### Trunk displacement

In the right direction, there was a significant difference between the interaction component Group^*^Position [*F*_(1, 149)_ = 5.77, *p* < 0.01]. When comparing the groups, the sitting position at 90° of flexion presented a lower trunk displacement in the late preterm group (*p* < 0.01; *d* = 1.32). There was a significant effect on the group factor [*F*_(1, 148)_ = 24.49, *p* < 0.01] and time in the midline [*F*_(2, 148)_ = 8.15, *p* < 0.01]. Lower trunk displacement was observed in the late preterm group and lower trunk displacement at 6 (*p* < 0.01; *d* = 0.76) and 7 months (*p* = 0.04; *d* = −1.10) compared to 8 months old.

However, in the right direction, there was a large effect size in the ring position at 7 and 8 months (*d* = 0.57, *d* = 0.66 respectively) and in the flexion position at 6, 7, and 8 months (*d* = 0.99, *d* = 1.73, *d* = 1.40, respectively). In the midline direction, there was a large effect size in the ring position at 6, 7, and 8 months (*d* = 0.89, *d* = 0.83, *d* = 0.97, respectively), and in the flexion position at 6, 7, and 8 months (*d* = 0.76, *d* = 1.22, *d* = 1.05, respectively) (Table 4).

## Discussion

The purpose of this study was to identify the level of trunk control and to investigate the influence of different sitting positions (ring and 90° of flexion) with accurate manual support in late preterm infants' trunks at a corrected age from 6 to 8 months old during reaching. We chose to study this age group, because in this period, infants are acquiring the ability to sit independently.

Using the SATCo, we found that generally younger infants at 6 months old presented trunk control in the upper, mid and lower thoracic regions. At 7 months old, they presented trunk control at the lower and upper lumbar thoracic levels; and older infants at 8 months old presented low lumbar trunk control and total trunk control. Our results corroborate with the study by van Der Fits et al. (9) who, by using electromyography, found that older infants showed more complex postural response patterns than younger infants, and that the order of recruitment of the trunk muscles occurred in the cephalocaudal direction (9). Similarly, Rachawani et al. (17, 25), using the SATCo, affirmed that full term infants developed trunk control in the cephalocaudal direction and in a segmental way (17, 25). The SATCo, therefore, is a precise assessment scale and can evaluate the trunk control in a segmental way.

It should be mentioned that our study provides important information regarding trunk control of late preterm infants at a corrected age, who despite having segmental trunk control in the cephalocaudal order, as full-term infants, they are delayed in terms of acquiring trunk control. Only 10% of the late preterm infants, even at a corrected age of 8 months, had total trunk control, while the majority of full-term infants (71.42%) already had total trunk control at this age. Our first hypothesis that late preterm infants had a lower level of trunk control than full-term infants was confirmed. We also suggest that a delay in the level of trunk control and acquiring independent sitting in late preterm infants is due to muscular hypotonia, a characteristic of this population, as stated by Plantinga et al. (12).

Regarding the influence of the ring sitting position and 90° of flexion on reaching, we found that there were no significant differences for most spatio and temporal variables for both groups and ages evaluated. Thus, we confirm our fourth hypothesis that providing accurate manual trunk support to late preterm infants and full term infants would not influence the spatio and temporal variables of reaching in the ring sitting position and sitting position at 90° of flexion. According to Van Der Fits et al. (9, 38), trunk stability induces a decrease in postural activity allowing infants to generate the torque needed to overcome gravity and perform reaching. Thus, it can be stated that the stability of postural control is a prerequisite for reaching (9, 38). The accurate trunk support we provided to the infants during the kinematic assessment of reaching promoted greater trunk stability in both sitting positions, and consequently, decreased the difficulties imposed by the organism and environment. Therefore, reaching was similar in the two sitting positions. We can reaffirm this idea by the results obtained from the study carried out by Silva et al. (26) who, by providing only manual pelvic support to full-term infants at 6 months old found that the ring sitting position and 90° of flexion influenced reaching performance (26).

We found that the maximum velocity to the right (peak velocity) during reaching was lower in the sitting position at 90° of flexion, indicating better reaching quality. In the midline, in the sitting position at 90° of flexion, it was less at 8 months compared to 6 months old, regardless of the group. This indicates that the infants performed better quality reaches when they had a lower level of trunk control. On the other hand, in the ring sitting position, the infants had a lower peak velocity in the midline at 6 months, regardless of the group, indicating that the ring sitting position facilitated better quality reaches even when the infants presented a higher level of trunk control in the sitting position, reinforcing the idea that a broader support base favors reaching in infants with a lower level of trunk control.

The sitting position at 90° of flexion influenced the trunk displacement to the right. The late preterm group presented less trunk displacement. This indicates that the late preterm group even at a corrected age presented less movement strategies, i.e., their trunk was more immobile during reaching in the position with less support base (90° of flexion). Corroborating with our study, Fallang et al. (16) also found that preterm infants without neurological lesions had immobile postural behavior during reaching at 4 months old in the supine position, and that the same behavior was not observed in full-term infants (16). Likewise, Kyvelidou et al. (2) found that preterm infants with delayed motor development reduced degrees of freedom to maintain stability in the sitting position (2). We suggest that the lower trunk mobility in the position at 90° of flexion is a strategy adopted by late preterm infants to perform reaching better. Therefore, training reaching in late preterm infants in this age group should be performed in the ring sitting posture.

When comparing the groups, concerning the spatio and temporal variables of reaching, our findings demonstrate that late preterm infants at a corrected age and accurate manual support in the trunk performed more immature reaches (more movement units and less rectitude index). In addition, at 6 months old, the straightness index was lower compared to 7 and 8 months old in both groups. These data indicate that over the months the trajectory of reaching became more rectilinear. The deceleration index to the right and left was higher in late preterm infants at a corrected age compared to full term infants, especially at 8 months old. Late preterm infants take longer to decelerate the movement of the upper limb before touching the target. Carvalho et al. (36) believe that by increasing the deceleration index, infants have more time to process and use visual information to touch the object (36). Compared to the full-term group, at 6 and 7 months old, the movement duration in the three directions was higher in the late preterm group, while the mean velocity in the same directions was less, even with the accurate manual support of the trunk. Corroborating with these data, Toledo and Tudella (13) found that late preterm infants presented slower reaches with lower maximum speed and more adjustments (13). We believe that slower-performing reaches are a strategy adopted by younger preterm infants to perform reaching better, minimizing typical intrinsic limitations such as poor eye coordination and reduced muscle tone.

Our data corroborate with Haddes-Algra and Carlberg (39) when she states that infants at 8 months old become more skilled concerning the disturbances imposed on them while carrying out tasks (39).

Our study provides valuable contributions because we longitudinally evaluate late preterm infants at ages considered key to the development of independent sitting. We provide these infants with accurate manual trunk support, following SATCo standards (firm and horizontal hands) so that they can perform reaching successfully, regardless of the position. Moreover, to the best of our knowledge, none of the studies found in the literature used SATCo in late preterm infants, which highlights the importance of our study in terms of understanding the development of trunk control in a segmental way in infants at risk. This is important for clinical practice, as knowing the exact level of trunk control can specifically intervene in the level of disability and make intervention more effective, resulting in better development of intervention strategies.

## Author contributions

NS and ET both contributed to the conception and design, acquisition, analysis, and interpretation of the data, drafting the article, critical revising of the intellectual content and the final approval of this study.

### Conflict of interest statement

The authors declare that the research was conducted in the absence of any commercial or financial relationships that could be construed as a potential conflict of interest.
